# Mortality risk estimation in acute calculous cholecystitis: beyond the Tokyo Guidelines

**DOI:** 10.1186/s13017-021-00368-x

**Published:** 2021-05-11

**Authors:** Ana María González-Castillo, Juan Sancho-Insenser, Maite De Miguel-Palacio, Josep-Ricard Morera-Casaponsa, Estela Membrilla-Fernández, María-José Pons-Fragero, Miguel Pera-Román, Luis Grande-Posa

**Affiliations:** 1 Department of Surgery, Autonomous University of Barcelona, Passeig Marítim de la Barceloneta, 25-29, 08003 Barcelona, Spain; 2 General Surgery Department, Hospital del Mar, Institut Hospital del Mar d’Investigacions Mèdiques (IMIM), Barcelona, Spain

**Keywords:** Acute cholecystitis, Acute calculous cholecystitis, Early cholecystectomy, High-risk patient, Delayed cholecystectomy, Percutaneous cholecystostomy, Non-surgical treatment, Mortality, Tokyo Guidelines, Charlson Comorbidity Index

## Abstract

**Background:**

Acute calculous cholecystitis (ACC) is the second most frequent surgical condition in emergency departments. The recommended treatment is the early laparoscopic cholecystectomy; however, the Tokyo Guidelines (TG) advocate for different initial treatments in some subgroups of patients without a strong evidence that all patients will benefit from them. There is no clear consensus in the literature about who is the unfit patient for surgical treatment. The primary aim of the study is to identify the risk factors for mortality in ACC and compare them with Tokyo Guidelines (TG) classification.

**Methods:**

Retrospective unicentric cohort study of patients emergently admitted with and ACC during 1 January 2011 to 31 December 2016. The study comprised 963 patients. Primary outcome was the mortality after the diagnosis. A propensity score method was used to avoid confounding factors comparing surgical treatment and non-surgical treatment.

**Results:**

The overall mortality was 3.6%. Mortality was associated with older age (68 + IQR 27 *vs.* 83 + IQR 5.5; *P* = 0.001) and higher Charlson Comorbidity Index (3.5 + 5.3 *vs.* 0+2; *P* = 0.001). A logistic regression model isolated four mortality risk factors (ACME): chronic obstructive pulmonary disease (OR 4.66 95% CI 1.7–12.8 *P* = 0.001), dementia (OR 4.12; 95% CI 1.34–12.7, *P* = 0.001), age > 80 years (OR 1.12: 95% CI 1.02–1.21, *P* = 0.001) and the need of preoperative vasoactive amines (OR 9.9: 95% CI 3.5–28.3, *P* = 0.001) which predicted the mortality in a 92% of the patients. The receiver operating characteristic curve yielded an area of 88% significantly higher that 68% (*P* = 0.003) from the TG classification. When comparing subgroups selected using propensity score matching with the same morbidity and severity of ACC, mortality was higher in the non-surgical treatment group. (26.2% *vs.* 10.5%).

**Conclusions:**

Mortality was higher in ACC patients treated with non-surgical treatment. ACME identifies high-risk patients. The validation to ACME with a prospective multicenter study population could allow us to create a new alternative guideline to TG for treating ACC.

**Trial registration:**

Retrospectively registered and recorded in Clinical Trials. NCT04744441

## Background

Early laparoscopic cholecystectomy is the recommended treatment for acute calculous cholecystitis (ACC) [1], but mortality in severe and/or fragile patients have promoted alternative initial non-surgical treatments (NST) such as percutaneous cholecystostomy (PC) or exclusive antibiotic treatment.

Regardless of the treatment modality, ACC does not have a high mortality rate, being 0.6% overall and 6% in severe cases [2–4] according to the highly influential Tokyo Guidelines (TG).

The optimal strategy for managing non-high-risk patients with ACC is laparoscopic cholecystectomy (LC), preferably carried out as soon as possible (2–3 days) [4–6]. There is however an active debate about the optimal timing for the cholecystectomy [7, 8]. A WSES recent analysis recommends the LC within 7 days from hospital admission and within 10 days from the onset of symptoms [1]. Nevertheless, despite the vast number of publications on ACC, a definite management strategy for extreme cases remains elusive. Small samples, heterogeneity in selection [9] and diagnosis, and assorted methodology flaws combine to obscure the definition of high-risk patients who may benefit from NST.

Using TG to dictate diagnosis, assessment, and treatment of ACC, it was expected that the vital prognosis would be greatly improved. Nevertheless, the mortality of ACC clusters in specific subgroups of patients where it is still significant. There is a crucial need to select the best strategy for these high-risk patients [10]. Regrettably, once the diagnostic of ACC is established, there is no worldwide consensus regarding who is a high-risk patient unfit for an urgent cholecystectomy. 

In the quest to define the high-risk patient, a collection of aggregate scores (APACHE [11], ASA [6, 12], P-POSSUM [12, 13], Charlson Comorbidity Index (CCI) [14], AAST [15], frailty score [16], and one or multiple organ dysfunctions [17] have been employed to predict mortality losing precision in the process and creating confusion of who is the patient unfit for surgery. The primary aim of the current study was to create a simpler and effective set of variables to identify high-risk patients isolating the discrete risk factors that predict mortality after ACC to select the patients best suited for NST.

## Methods

This was a retrospective study carried out in a single center with a dedicated surgical emergency unit, from January 2011 to December 2016, in a Metropolitan University Hospital in Barcelona, Spain. The data were reviewed and completed by 2018, and the analysis was completed in March 2020.

The study candidates comprised 963 consecutive patients with a diagnosis of acute cholecystitis.

### Inclusion and exclusion criteria

**Fig. 1:**
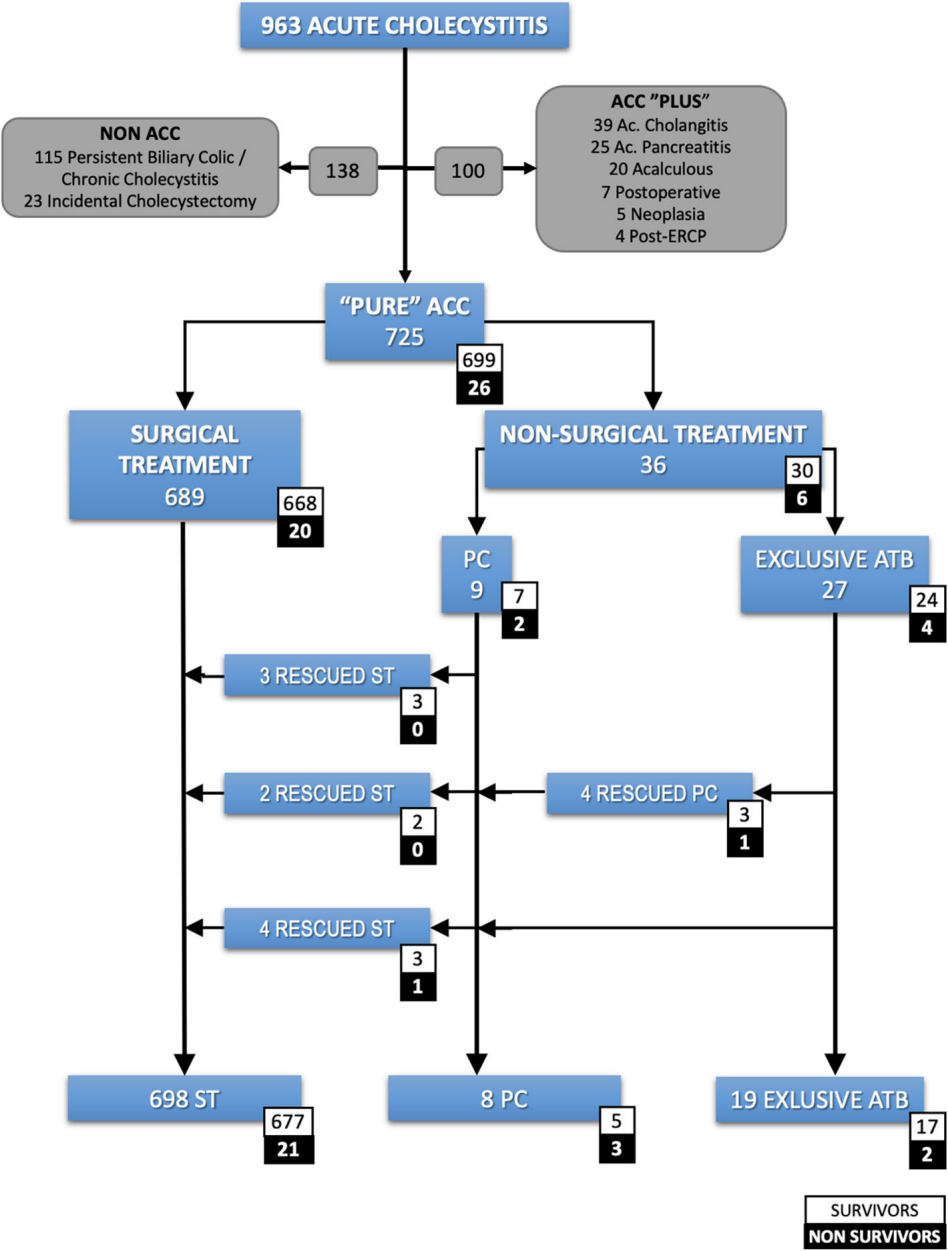
Patient selection and patient flow diagram

All patients were selected if they had acute cholecystitis according to the Tokyo Guidelines of 2018 (TG18) and/or received a diagnosis of ACC in the Pathology report (Table 1). The study case definition was a ‘pure acute cholecystitis’; therefore, patients with any other concomitant diagnosis potentially influencing outcome (postoperative cholecystitis, acute cholangitis, acute pancreatitis, and post-endoscopic retrograde pancreato-cholangiography, or neoplasia) were excluded from the final analysis (Fig. 1).

**Table 1:** Differences between survivors and non-survivors in pre-treatment and post-treatment variables

**Variable**		**Odds ratio**^b^	^**95%**^ **IC**	***P***
**Gender** (M *vs*. F)		0.898	0.41–1.97	0.791
**ASA score**^c^	II	1.03	0.99–1.08	0.666
	III	1.08	1.04–1.12	0.019
	IV	1.21	1.04–1.42	0.001
**Tokyo International Guidelines Severity Grading**^d^ **(TGSG)**	II (moderate)	3.19	0.38–26.78	0.258
	III (severe)	10.3	1.36–77.7	0.005
**TGSG PAFI** < 300		0.39	0.042–3.58	0.390
**TGSG Oliguria** (diuresis < 0.5 mL/kg/h)		18.19	7.47–44.29	0.001
**TGSG marked local inflammation**		2.26	0.811–6.29	0.109
**TGSG WBC** > 18,000/mm^3^		3.73	1.63–8.52	0.001
**TGSG PT-INR** > 1.5		3.83	1.6–9	0.001
**TGSG renal dysfunction** (creatinine> 2 mg)		12.65	5.58–28.65	0.001
**TGSG neurological dysfunction**		1.65	0.67–4.06	0.269
**TGSG cardiovascular dysfunction** (amines)		18.94	7.44–48.18	0.001
**TGSG Murphy’s sign**		0.5	0.23–1.11	0.082
**TGSG palpable tender mass in RUAQ**		1.4	0.62–3.4	0.399
**TGSG tachypnea** (> 20 bpm)		9.46	4–22.4	0.001
**TGSG duration of complaints > 72 h**		2.15	0.96–4.82	0.056
**SIRS**		10.21	3.45–30.27	0.001
		**Exitus**^a^ ***N*** **= 26 (3.6)**	**Survivors**^a^ ***N*** **= 699 (96.4)**	6. ***P***
**Charlson Comorbidity Index**		3.50 (5.3)	0 (2)	0.001
**Age** (years)		83.0 (5.5)	68.0 (27)	0.001
**Bilirubin** (mg/dL)		1.64 (0.9)	0.86 (1.1)	0.002
**Creatinine** (gr/dL)		1.7 (2.2)	0.80 (0.4)	0.001
**Alkaline phosphatase** (UI/L)		142 (152)	93 (72)	0.001
**Gamma-glutamyl-transpherase** UI/L)		71 (71)	123 (239)	0.317
**Glutamil oxaloacetic transaminase** (UI/L)		57 (167)	27 (41)	0.005
**PT-INR**		1.43 (0.3)	1.19 (0.2)	0.001
**Lactate** (gr/dL)		2.20 (3)	1.5 (1.1)	0.008
**Temperature** (°C)		37.0 (1.7)	36.3 (1)	0.289
**WBC** > 1000/mm^3^		17 (13)	14 (7)	0.016
**Partial oxygen pressure (mmHg)**		98 (10)	99 (1)	0.010
**CRP** (gr/dL)		23.7 (22)	14 (26)	0.110
**Platelets** (1000/mm^3^)		162 (162)	210 (129)	0.210
**Treatment**				
	***N*** **(%)**	**OR**	^**95%**^ **IC**	7. ***P***
**Cholecystectomy as first treatment**	689 (95)	0.149	0.056–0.399	0.001
**Cholecystectomy as last treatment**	9 (1.2)	4.181	0.499–35.041	0.242
**Laparotomy as initial approach**	75 (10)	25.708	9.618–68.71	0.001
**Converted laparoscopy**	93 (13)	0.678	0.155–2.959	1.000
**Additional procedures**	164 (23)	3.087	1.28–7.40	0.015
**Cholecystostomy as first treatment**	9 (1.2)	1.643	0.247–10.95	0.627
**Cholecystostomy as rescue treatment**	4 (0.5)	1.800	0.154–20.99	0.535
**Antibiotics as primary treatment**	27 (3.7)	0.609	0.091–4.056	0.627
**Post-treatment**				
		**Exitus** ***N*** **= 26 (3.6)**	**Survivors** ***N*** **= 699 (96.4)**	8. ***P***
**Reoperation**		0 (0)	2 (0.3)	1.000
**Postoperative hospital stay** (days)		13 (19)	3 (5)	0.001
**Postoperative complications**		26 (100)	287 (41)	0.001
**Postoperative complications** **(Clavien-Dindo)**	**No**		412 (59)	
	**I**		105 (15)	
	**II**		88 (12.6)	
	**IIIa**		45 (6.4)	
	**IIIb**		17 (2.4)	
	**IVa**		16 (2.3)	
	**IVb**		15 (2.1)	
	**Severe**^**e**^		48 (7)	

*PAFI* Pa0_2_/Fi0_2_, *WBC* white cells blood count, *PT-INR* prothrombin time international normalized ratio, *SIRS* systemic inflammatory response syndrome ≥ 2 points, *RUAQ* right upper abdominal quadrant, *bpm* breaths per minute, *CPR* C-reactive protein

^a^*N* (%) or median (IQR)

^b^ Odds ratio for mortality

^c^ASA score 1 has been used to calculate the odds ratio for the remaining groups; ASA score V is not reported as there was only one patient in this group

^d^Tokyo Guidelines Classification Year 2013 [18] TG I was used to calculate the odds ratio for the remaining groups

^e^> Clavien-Dindo I

### Variables

Primary data were available from a prospective database maintained in File Maker v.12 (Mountainview, CA, USA), which included basic demographic data, type of interventions, sex, total days of admission, and complications. Every record was completed by browsing the electronic patient record, adding laboratory, and microbiology data, as well as antibiotic therapy, duration of procedure, additional procedures, and grade of acute cholecystitis according to the TG18 diagnostic criteria (Table 1).

Preoperative comorbidities were assessed using the CCI [14] and surgical risk by ASA classification [19]. The type of initial treatment was classified as surgical treatment (ST; cholecystectomy either by laparoscopy or laparotomy) or NST, which was either percutaneous cholecystostomy (PC) or intravenous antibiotics alone.

The main outcome measure was the mortality after the diagnostic of ACC (30 days if the patient is discharged from the hospital or at any time during the same admission if not discharged).

### Interventions

All patients received intravenous antibiotic therapy as soon as the diagnosis was formulated, according to a fixed protocol.

Ultrasound-guided cholecystostomy was performed percutaneously with an 8-Fr catheter (SKATER ™, Argon Medical Devices, Rochester, NY, USA) by either transhepatic or transperitoneal insertion, at the discretion of the radiologist.

LC was performed according to the French technique using 4 trocars. The content of the gallbladder was evacuated by Veress needle puncture when necessary.

### Statistical analysis

The normal distribution of the quantitative variables was assessed using the Kolmogorov–Smirnov test, which showed that none of the variables were normally distributed; therefore, their values were expressed as median and interquartile ranges. The Mann–Whitney *U* non-parametric test was used to assess the significance of differences between means.

The association between qualitative variables was assessed with the chi-square test or Fisher’s exact test, as required. The increased risk of an event associated with a variable was reported as the odds ratio (OR) and 95% confidence interval (CI).

Additionally, as this was a retrospective observational study and the treatment groups were markedly asymmetric, we used the propensity score matching method [20] to select and compare two subgroups of patients evenly balanced by severity according to the TG18 criteria and by comorbidity according to the CCI.

A model for predicting mortality was built using binomial logistic regression with stepwise progressive conditional entry and standard baseline conditions for admission and rejection of variables with significant differences in the univariate analysis. The discrimination power of the model was assessed by receiver operating characteristic (ROC) curves and was compared with the DeLong methods.

## Results

This study was based on a group of 963 patients with acute cholecystitis, from whom 725 patients with pure ACC were selected (Fig. 1). Of these, 689 underwent initial ST and 36 (5%) underwent NST. Among ST patients, the median time from onset of symptoms to surgery was 3 days (^25–75^IQR 2–5). Among the NST patients, 27 (75%) initially received only antibiotics and 9 (25%) received PC as primary approach. Subsequently, 4 (15%) patients of the only antibiotics initial treatment received PC due to unfavorable evolution, totaling 13 (36%) patients with PC, 5 (38%) of whom were finally cholecystectomized owing to a worsening clinical course (three of them were initially treated with a primary PC and two were a secondary PC after a failure of exclusive antibiotics treatment). Overall, 698 (96.3%) urgent cholecystectomies were performed.

The median age of patients was 69 years (interquartile range, 53–80 years), and the elderly (≥ 80 years) represented 26% of the total.

The prevalence of diabetes mellitus and heart failure was noteworthy, followed by kidney disease, peripheral vascular disease, chronic obstructive pulmonary disease (COPD), and acute myocardial infarction. Most of the patients were classified as ASA II (52.3%) or ASA III (32.8%) (Table 1).

The grade of cholecystitis according to TG18 was mild in 21%, moderate in 39%, and severe in 40% of patients. The severity factors for each group are shown in Table 1.

In 689 (95%) patients, ST was initially indicated, which was by laparotomy in 75 (11%), and laparoscopic in 623 (89%) of which 93 (13%) were converted to open cholecystectomy.

### Mortality

Overall, the mortality rate of the series was 3.6%. The mortality of each treatment type, including treatments applied as rescue, is detailed in Fig. 1.

Legend: ACC: acute calculous cholecystitis. ST: surgical treatment. NST: non-surgical treatment. PC: percutaneous cholecystostomy. ATB: antibiotics

**Fig. 2:**
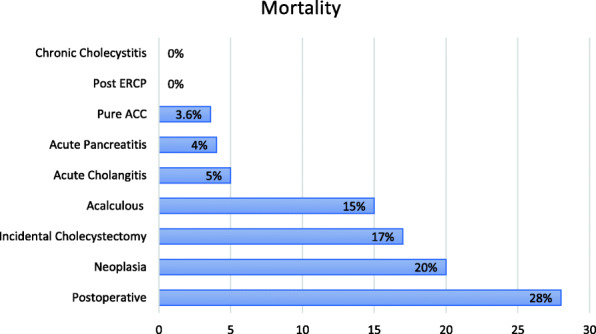
Mortality depending on the precise etiology of ACC

The patients excluded from the analysis because they developed a non-pure ACC exhibited a wide variation in mortality rates (from 0 to 28%) depending on the precise etiology (Fig. 2).

**Fig. 3:**
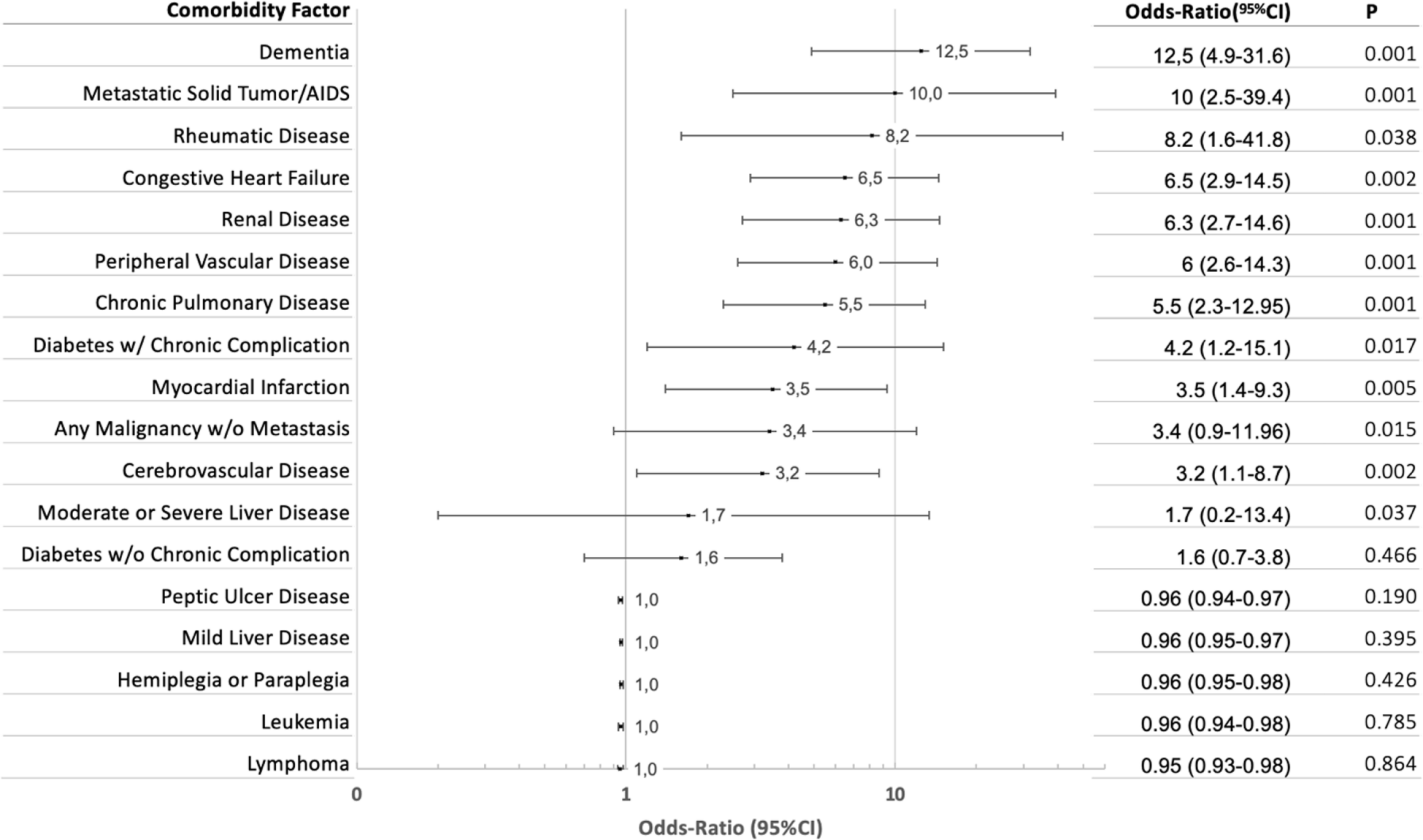
Charlson Comorbidity Index components as risk for mortality in acute cholecystitis

Patients who died were almost 20 years older, had higher ASA scores and CCI, and had the same distribution of individual comorbidities (Table 1). Each discrete comorbidity factor of the CCI carried a disparate relative risk for mortality (OR from 1 to 12), as depicted in Fig. 3.

The mortality rate of patients with severe cholecystitis (grade III of the TG18) was nine times greater than that of patients with mild cholecystitis. The clinical and biological variables of patients with associated ORs for mortality are detailed in Table 1.

Patients with an initial NST experienced a mortality six times higher than those with initial ST. Differences in mortality between patients initially ascribed to exclusive antibiotics or PC were not significant (15% vs. 22%; *P* = 0.62). The open cholecystectomy approach was followed by a 20-fold higher mortality than the laparoscopic cholecystectomy approach (20% vs. 1%; *P* = 0.001). However, the laparoscopic converted procedures had similar mortality than non-converted cholecystectomies (2.2% vs 3.1%; *P* = 1). Patients who switched from NST to ST (*n* = 9) had an overall mortality of 11%.

### Propensity score matching: comparison among subgroups of similar severity

From the 689 patients with ST, a subgroup of 36 patients with ACC was pair-matched for identical severity (TG18) to the 36 patients with NST using the propensity score matching method. Mortality in the NST group was twice that of the ST group, although this difference was not statistically significant owing in part to the low prevalence (Table 2).

**Table 2:** Differences between iST and iNST with the PSM analysis

Variable		iST^a^(19)	iNST^a^ (19)	***P***
**Complications**		63.2% (12)	63.2% (12)	0.631
**Severe complications**^b^		26.3% (5)	42.1% (8)	0.248
**Postoperative complications (Clavien-Dindo)**	**No**	36.8% (7)	36.8% (7)	
	**Grade I**	10.5% (2)	5.3% (1)	
	**Grade II**	15.8% (3)	5.3% (1)	
	**Grade IIIa**	10.5% (2)	10.5% (2)	
	**Grade IIIb**	5.3% (1)	10.5% (2)	
	**Grade IV**	10.5% (2)	5.3% (1)	
	**Grade V**	10.5% (2)	26.5% (5)	

^a^% (patients). *iST* initial surgical treatment, *iNST* initial non-surgical treatment

^b^Clavien-Dindo classification > IIIb

### Predictive model

**Fig. 4:**
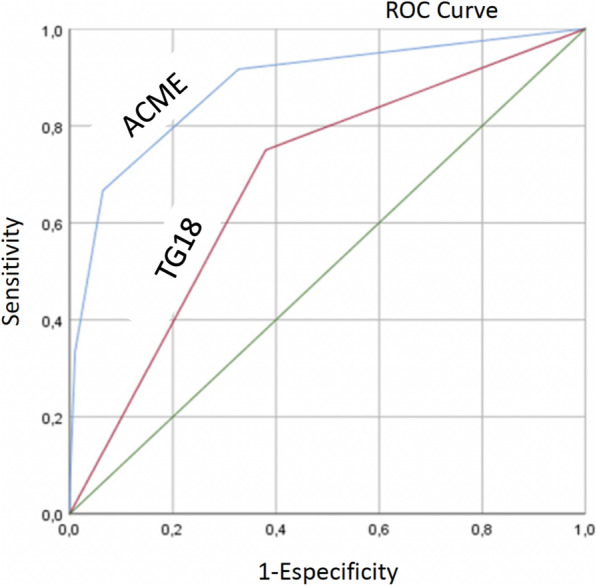
ROC curve for mortality: ACME vs TG18

A multivariate model predicting mortality, the acute cholecystitis mortality estimation (ACME), retained a set of four variables: COPD (OR 4.66; 95% CI 1.7–12.8; *P* = 0.001), dementia (OR 4.12; 95% CI 1.34–12.7; *P* = 0.001), age > 80 years (OR 1.12; 95% CI 1.02–1.21; *P* = 0.001), and preoperative vasoactive amines (OR 9.9; 95% CI 3.5–28.3; *P* = 0.001), which accurately predicted mortality in 92% of cases. The ROC curve yielded an area under the curve of 88%, well above the 68% (*P* = 0.003) from the TG18 classification (Fig. 4).

## Discussion

A distinctive feature of this study is the strict selection of patients. Some 238 patients were discarded to obtain a homogeneous cohort of pure AAC [21, 22]. Many source papers used by the TG18 suffer from multiple etiologies pooling [3, 21–23] or only exclude the chronic cholecystitis subgroup [24], undoubtedly being a source of statistical noise. Some reports on ACC perform some intents of refined patient selection [25], but a strict approach has not been used before, and we believe it is essential.

The current study reports on the experience meticulously registered in a surgical emergency unit where NST has not been adopted as recommended by TG18. In fact, we opted for NST in 5% of patients, whereas 40% of the series were classified as TG grade III. This attitude has been adopted by a significant number of groups with ample clinical experience in ACC management [15, 26–29].

In the current series, the distributions of TG18 severity classification were clearly more slanted toward severity than the majority of other series of non-selected ACC where the severe proportion oscillates between 5 and 19% [23, 29–31]. This plethora of severe is mainly due to higher proportions of kidney and neurological dysfunction, and/or international normalized ratio ≥ 1.5 among our patients.

### Mortality

According to the TG18, the accepted mortality for acute cholecystitis should be < 1% [4]. In the current study, the 30-day mortality was substantially higher (3.6%). Large multicenter studies reported a mortality rate from 0.6 to 13.5% [23, 30, 32, 33]. Nevertheless, a fifth of the 26 patients who died in our series did so after the standardized 30 days to calculate postoperative mortality. The mortality rate not including these patients would drop to 2.8%. Furthermore, if chronic cholecystitis and post-endoscopic retrograde cholangiopancreatography cases were included in the total count, as is routinely done in many series, the mortality would have been wrongly reported at 1.8%, missing almost half of the deceased due to complications after ACC was diagnosed and treated. In contrast, our series did not include acalculous or postoperative cholecystitis; two etiologies with very low prevalence but higher mortality and morbidity [18].

Advanced age, high ASA score, and CCI are almost universally cited as mortality risk factors, not only for ACC but for any emergency procedure [23, 34, 35]. However, not every discrete comorbidity factor of the CCI carries the same relative risk for mortality, their ORs ranging from 1 to 12, as depicted in Fig. 3. That is why we chose to “deconstruct” the CCI and calculate the contribution of each component of the specific population of ACC patients.

### Identifying the high-risk patient

Regarding reducing mortality, surgeons have been struggling to identify patients too frail and/or too severely affected, who will be better served without surgery. In this context, we label this patient as a high-risk patient. The standardization and use of therapeutic algorithms that include the preoperative assessment of surgical risk mortality rate of patients admitted with a diagnosis of acute cholecystitis is essential. Hypothetically, in some high-risk patients at admission, their general condition may be improved with medical treatment, and a risk evaluation carried out 24–48 h after admission could reassign the patient to the group of early ST.

Some authors consider high-risk patients as those with failure of at least one organ or multiple organ dysfunctions [36]. Patients with an ASA III or IV score have an expected postoperative mortality of 5–27%, are considered high risk for cholecystectomy [37], and have higher morbidity [23, 38]. In contrast, in a prospective observational study, González-Muñoz et al. found that patients with ASA > II and only medical treatment had a mortality of 17%, whereas those operated upon early had no mortality [13, 24]

In the current series, nearly 40% of patients would have been labeled as high risk. However, we believe that we should not exclude them from early surgery.

Age alone appears in some studies as an independent surgical risk [39]. However, age by itself does not increase mortality [40]. Decreased functional reserve capacity in addition to comorbidities, usually but not always linked to age, are the main risk factors for mortality [39]. Consequently, although age per se is not regarded as a contraindication for early cholecystectomy, it has been widely recognized that the use of frailty and surgical risk scores could contribute to achieving the best clinical judgment in elderly people [40].

There is no consensus on which of the already available surgical risk scores better predicts postoperative mortality in acute cholecystitis. APACHE II > 15 [41], CCI > 6 [23], and P-POSSUM > 40 [12, 13] have all been used to support ST. None of them were specifically created for ACC; therefore, none of them are particularly advantageous over the others, and none of them offer an outstanding prediction ability. A variety of frailty scores have been widely used to predict surgical outcomes in vast register-based studies [42]. Few studies have assessed the influence of frailty on outcomes for patients with CAL. Fagenson et al. selected the modified frailty index to successfully predict both mortality (AUC = 0.83) and Clavien-Dindo grade IV complications (AUC = 0.73) in a NSQIP-based study. Both ACME and frailty coincide in including COPD and dementia as a strong predictors for mortality [16].

We found that the individual weight of each variable included in the CCI was quite different when studied in a cohort of ACC patients (Fig. 3).

### Acute cholecystitis mortality estimation

By selecting variables with a greater impact on the univariate analysis, we devised the acute cholecystitis mortality estimation (ACME). The model retained a set of four variables which accurately predicted mortality better than the TG classification (Fig. 4). Using a similar approach, Fagenson et al. chose the modified frailty index to identify patients with the worst prognosis after cholecystectomy. They also opted for extracting the more significant CCI components and ended up with a very similar AUC of 83% for predicting mortality [16].

### Treatment modalities

Patients with initial NST experienced a mortality six times higher than those initially selected for initial ST. Differences in mortality between patients initially ascribed to exclusive antibiotic therapy or cholecystostomy were not significant. Patients who switched from NST to ST (*n* = 9) had an overall mortality of 11%. Likewise, a massive observational prospective study by Endo et al. compared four strategies (antibiotic therapy, cholecystostomy either as a definitive treatment or as a bridge to surgery, and early cholecystectomy). Their results supported early cholecystectomy over any other strategy, with or without cholecystostomy [38]. ST is the first option in the management of ACC crystallized in the CHOCOLATE trial, which encouraged early laparoscopic cholecystectomy over cholecystostomy [41].

The complexity of the course of treatment, with subgroups of NST patients requiring rescue cholecystostomy and those in turn that finally required rescue cholecystectomy is reflected in Fig. 1. In every non-randomized trial, there are patients migrating from the four initial treatment groups (to be analyzed by intention-to-treat) to treatments finally received (to be analyzed per protocol). This circumstance undoubtedly contributed to the long period of uncertainty surrounding the best therapy option for high-risk patients with ACC.

### Propensity score matching: comparison among subgroups of similar severity

This technique has been used occasionally in the cholecystectomy series [43, 44]. In the current study, when comparing high-risk subgroups with the same preoperative morbidity and ACC severity selected by PSM, the mortality was higher in the NST group than in the ST group (26.2% *vs.* 10.5%), probably reflecting that we reserved the NST for patients with more severe ACC.

### Limitations of the study

The retrospective nature of this study is undoubtedly its main limitation. In contrast, the limited application of NST makes this cohort valid to determine risk factors for postoperative complications but less solvent when determining the best strategy for severe ACC in the common range for a series of consecutive patients in a single center [23, 41].

The patients in this study had more advanced disease, the severe ACC is being overrepresented in comparison with most other series which can bias the conclusions toward a higher mortality estimation. However, we believe that this population composition makes this analysis more robust in assessing risk factors for mortality.

The follow-up was limited to 30 days after the intervention or until the patient's discharge. More extensive monitoring would likely discover a greater number of complications [13]. Nevertheless, we reported mortality directly related to the ACC taking place beyond the 30th day of admission, which is routinely missing from other reports circumscribed to 30-day mortality.

## Conclusion

Laparoscopic cholecystectomy is the recommended treatment for acute calculous cholecystitis, but not all patients benefit from initial surgical treatment.

The Tokyo Guidelines classification would probably be as effective using only 2 groups: mild (mild and moderate) and severe ACC.

Not all the factors of Charlson Comorbidity Index carry the same risk of mortality in ACC. A new simplified, highly predictive model of mortality (ACME) included a different set of variables that included age > 80 years, COPD, dementia, and administration of amines in the preoperative period.

In line with the principal aim of the study, the mortality risk score ACME could promptly identify the high-risk patient with ACC in our population. Its validation comparing it with the TG in a prospective multicenter study is now mandatory.

## Data Availability

The datasets generated and analyzed during the current study are available from the corresponding author on reasonable request, but the results are going to be able to consult in ClinicalTrials.gov for a year.

## Abbreviations

ACC: Acute calculous cholecystitis
ST: Surgical treatment
NST: Non-surgical treatment
PC: Percutaneous cholecystostomy
ATB: Antibiotics
CCI: Charlson Comorbidity Index
ASA: American Society Anesthesiologist score
TG: Tokyo Guidelines
OR: Odds ratio
CI: Confidence interval
ROC: Receiver operating characteristic
IQR: Interquartile range
COPD: Chronic obstructive pulmonary disease
AMI: Acute myocardial infarction

## References

[1] Pisano M, Allievi N, Gurusamy K, Borzellino G, Cimbanassi S, Boerna D, Coccolini F, Tufo A, di Martino M, Leung J, Sartelli M, Ceresoli M, Maier RV, Poiasina E, de Angelis N, Magnone S, Fugazzola P, Paolillo C, Coimbra R, di Saverio S, de Simone B, Weber DG, Sakakushev BE, Lucianetti A, Kirkpatrick AW, Fraga GP, Wani I, Biffl WL, Chiara O, Abu-Zidan F, Moore EE, Leppäniemi A, Kluger Y, Catena F, Ansaloni L (2020). 2020 World Society of Emergency Surgery updated guidelines for the diagnosis and treatment of acute calculus cholecystitis. World J Emerg Surg..

[2] Shaffer EA (2005). Epidemiology and risk factors for gallstone disease: has the paradigm changed in the 21st century?. Curr Gastroenterol Rep..

[3] Yokoe M, Takada T, Hwang TL, Endo I, Akazawa K, Miura F, Mayumi T, Mori R, Chen MF, Jan YY, Ker CG, Wang HP, Itoi T, Gomi H, Kiriyama S, Wada K, Yamaue H, Miyazaki M, Yamamoto M (2017). Validation of TG13 severity grading in acute cholecystitis: Japan-Taiwan collaborative study for acute cholecystitis. J Hepatobiliary Pancreat Sci..

[4] Okamoto K, Suzuki K, Takada T, Strasberg SM, Asbun HJ, Endo I, Iwashita Y, Hibi T, Pitt HA, Umezawa A, Asai K, Han HS, Hwang TL, Mori Y, Yoon YS, Huang WSW, Belli G, Dervenis C, Yokoe M, Kiriyama S, Itoi T, Jagannath P, Garden OJ, Miura F, Nakamura M, Horiguchi A, Wakabayashi G, Cherqui D, de Santibañes E, Shikata S, Noguchi Y, Ukai T, Higuchi R, Wada K, Honda G, Supe AN, Yoshida M, Mayumi T, Gouma DJ, Deziel DJ, Liau KH, Chen MF, Shibao K, Liu KH, Su CH, Chan ACW, Yoon DS, Choi IS, Jonas E, Chen XP, Fan ST, Ker CG, Giménez ME, Kitano S, Inomata M, Hirata K, Inui K, Sumiyama Y, Yamamoto M (2018). Tokyo Guidelines 2018: flowchart for the management of acute cholecystitis. J Hepatobiliary Pancreat Sci..

[5] Zafar SN, Obirieze A, Adesibikan B, Cornwell EE, Fullum TM, Tran DD (2015). Optimal time for early laparoscopic cholecystectomy for acute cholecystitis. JAMA Surg..

[6] Gutt CN, Encke J, Köninger J, Harnoss JC, Weigand K, Kipfmüller K, Schunter O, Götze T, Golling MT, Menges M, Klar E, Feilhauer K, Zoller WG, Ridwelski K, Ackmann S, Baron A, Schön MR, Seitz HK, Daniel D, Stremmel W, Büchler MW (2013). Acute cholecystitis: early versus delayed cholecystectomy, a multicenter randomized trial (ACDC Study, NCT00447304). Ann Surg..

[7] Kao LS, Ball CG, Chaudhury PK (2018). Evidence-based reviews in surgery. early cholecystectomy for cholecystitis. Ann Surg..

[8] Macafee DA, Humes DJ, Bouliotis G, Beckingham IJ, Whynes DK, Lobo DN (2009). Prospective randomized trial using cost-utility analysis of early versus delayed laparoscopic cholecystectomy for acute gallbladder disease. Br J Surg..

[9] Campanile FC, Catena F, Coccolini F, Lotti M, Piazzalunga D, Pisano M, Ansaloni L (2011). The need for new “patient-related” guidelines for the treatment of acute cholecystitis. World J Emerg Surg..

[10] Pisano M, Campanile FC. Acute calculus cholecystitis: commentary on Tokyo Guidelines 2018. J Hepatobiliary Pancreat Sci. 2018;25(3):E3–4. 10.1002/jhbp.535. Accessed 2011. 10.1002/jhbp.535 29532639

[11] Kortram K, van Ramshorst B, Bollen TL, Besselink MGH, Gouma DJ, Karsten T, Kruyt PM, Nieuwenhuijzen GAP, Kelder JC, Tromp E, Boerma D (2012). Acute cholecystitis in high risk surgical patients: Percutaneous cholecystostomy versus laparoscopic cholecystectomy (CHOCOLATE trial): Study protocol for a randomized controlled trial. Trials..

[12] The Royal College of Surgeons of England/Department of Health. The Higher Risk General Surgical Patient: Towards Improved Care for a Forgotten Group. London; 2011. https://www.rcseng.ac.uk/library-and-publications/rcs-publications/docs/the-higher-risk-general-surgical-patient/.

[13] González-Muñoz JI, Franch-Arcas G, Angoso-Clavijo M, Sánchez-Hernández M, García-Plaza A, Caraballo-Angeli M, Muñoz-Bellvís L (2017). Risk-adjusted treatment selection and outcome of patients with acute cholecystitis. Langenbeck’s Arch Surg..

[14] Charlson ME, Pompei P, Ales KL, MacKenzie R, MacKenzie CR (1987). A new method of classifying prognostic comorbidity in longitudinal studies: Development and validation. J Chronic Dis.

[15] Hernández M, Murphy B, Aho JM (2018). Validation of the AAST EGS acute cholecystitis grade and comparison with the Tokyo guidelines. Surgery..

[16] Fagenson AM, Powers BD, Zorbas KA, Karhadkar S, Karachristos A, di Carlo A, Lau KN (2020). Frailty predicts morbidity and mortality after laparoscopic cholecystectomy for acute cholecystitis: an ACS-NSQIP cohort analysis. J Gastrointest Surg..

[17] Resio BJ, Chiu AS, Zhang Y, Pei KY (2020). Characterization of high mortality probability operations at national surgical quality improvement program hospitals. JAMA Surg..

[18] Treinen C, Lomelin D, Krause C, Goede M, Oleynikov D (2015). Acute acalculous cholecystitis in the critically ill: risk factors and surgical strategies. Langenbeck’s Arch Surg..

[19] Mayhew D, Mendonca V, Murthy BVS (2019). A review of ASA physical status – historical perspectives and modern developments. Anaesthesia..

[20] Austin PC (2011). An introduction to propensity score methods for reducing the effects of confounding in observational studies. Multivariate Behav Res..

[21] Yokoe M, Takada T, Hwang TL, Endo I, Akazawa K, Miura F, Mayumi T, Mori R, Chen MF, Jan YY, Ker CG, Wang HP, Itoi T, Gomi H, Kiriyama S, Wada K, Yamaue H, Miyazaki M, Yamamoto M (2017). Descriptive review of acute cholecystitis: Japan-Taiwan collaborative epidemiological study. J Hepatobiliary Pancreat Sci..

[22] Naidu K, Beenen E, Gananadha S, Mosse C (2016). The yield of fever, inflammatory markers and ultrasound in the diagnosis of acute cholecystitis: a validation of the 2013 Tokyo Guidelines. World J Surg..

[23] Endo I, Takada T, Hwang TL, Akazawa K, Mori R, Miura F, Yokoe M, Itoi T, Gomi H, Chen MF, Jan YY, Ker CG, Wang HP, Kiriyama S, Wada K, Yamaue H, Miyazaki M, Yamamoto M (2017). Optimal treatment strategy for acute cholecystitis based on predictive factors: Japan-Taiwan multicenter cohort study. J Hepatobiliary Pancreat Sci..

[24] Yokoe M, Takada T, Strasberg SM, Solomkin JS, Mayumi T, Gomi H, Pitt HA, Gouma DJ, Garden OJ, Büchler MW, Kiriyama S, Kimura Y, Tsuyuguchi T, Itoi T, Yoshida M, Miura F, Yamashita Y, Okamoto K, Gabata T, Hata J, Higuchi R, Windsor JA, Bornman PC, Fan ST, Singh H, de Santibanes E, Kusachi S, Murata A, Chen XP, Jagannath P, Lee S, Padbury R, Chen MF, Tokyo Guidelines Revision Committee (2012). New diagnostic criteria and severity assessment of acute cholecystitis in revised Tokyo guidelines. J Hepatobiliary Pancreat Sci..

[25] Lin D, Wu S, Fan Y, Ke C (2020). Comparison of laparoscopic cholecystectomy and delayed laparoscopic cholecystectomy in aged acute calculous cholecystitis: a cohort study. Surg Endosc..

[26] Bekki T, Abe T, Amano H, Hanada K, Kobayashi T, Noriyuki T, et al. Validation of the Tokyo guideline 2018 treatment proposal for acute cholecystitis from a single-center retrospective analysis. Asian J Endosc Surg. 2020(1):1–7. 10.1111/ases.12801. 10.1111/ases.12801 32285589

[27] Abe T, Amano H, Hanada K, Bekki T, Minami T, Yonehara S, Noriyuki T, Nakahara M (2019). Efficacy and safety of early cholecystectomy for comorbid acute cholecystitis and acute cholangitis: Retrospective cohort study. Ann Med Surg..

[28] Pisano M, Ceresoli M, Allegri A (2015). Single center retrospective analysis of early vs. delayed treatment in acute calculous cholecystitis: application of a clinical pathway and an economic analysis. Ulus Travma Acil Cerrahi Derg..

[29] Amirthalingam V, Low JK, Woon W, Shelat V (2017). Tokyo Guidelines 2013 may be too restrictive and patients with moderate and severe acute cholecystitis can be managed by early cholecystectomy too. Surg Endosc Other Interv Tech..

[30] Lee S-W, Yang SS, Chang C-S, Yeh H-J (2009). Impact of the Tokyo guidelines on the management of patients with acute calculous cholecystitis. J Gastroenterol Hepatol..

[31] Joseph B, Jehan F, Dacey M (2018). Evaluating the relevance of the 2013 Tokyo Guidelines for the diagnosis and management of cholecystitis. J Am Coll Surg..

[32] Lo C-M, Liu C-L, Fan S-T, Glas F, Lai EC, Wong J (1998). Prospective randomized study of early versus delayed laparoscopic cholecystectomy for acute cholecystitis. Ann Surg..

[33] Rice CP, Vaishnavi KB, Schaeffer AB (2019). Operative complications and economic outcomes of cholecystectomy for acute cholecystitis. World J Gastroenterol..

[34] Strasberg SM (2008). Acute Calculous Cholecystitis. N Engl J Med..

[35] Bonaventura A, Leale I, Carbone F, Liberale L, Dallegri F, Montecucco F, Borgonovo G (2019). Pre-surgery age-adjusted Charlson Comorbidity Index is associated with worse outcomes in acute cholecystitis. Dig Liver Dis..

[36] Yokoe M, Takada T, Strasberg SM, Solomkin JS, Mayumi T, Gomi H, Pitt HA, Garden OJ, Kiriyama S, Hata J, Gabata T, Yoshida M, Miura F, Okamoto K, Tsuyuguchi T, Itoi T, Yamashita Y, Dervenis C, Chan AC, Lau WY, Supe AN, Belli G, Hilvano SC, Liau KH, Kim MH, Kim SW, Ker CG, Tokyo Guidelines Revision Committee (2013). TG13 diagnostic criteria and severity grading of acute cholecystitis (with videos). J Hepatobiliary Pancreat Sci..

[37] Feigal DW, Blaisdell FW (1979). The estimation of surgical risk. Med Clin North Am..

[38] Anderloni A, Buda A, Vieceli F, Khashab MA, Hassan C, Repici A (2016). Endoscopic ultrasound-guided transmural stenting for gallbladder drainage in high-risk patients with acute cholecystitis: a systematic review and pooled analysis. Surg Endosc..

[39] Saunders DI, Murray D, Pichel AC, Varley S, Peden CJ (2012). Variations in mortality after emergency laparotomy: The first report of the UK emergency laparotomy network. Br J Anaesth..

[40] Pisano M, Ceresoli M, Cimbanassi S, Gurusamy K, Coccolini F, Borzellino G, Costa G, Allievi N, Amato B, Boerma D, Calcagno P, Campanati L, Campanile FC, Casati A, Chiara O, Crucitti A, di Saverio S, Filauro M, Gabrielli F, Guttadauro A, Kluger Y, Magnone S, Merli C, Poiasina E, Puzziello A, Sartelli M, Catena F, Ansaloni L (2019). 2017 WSES and SICG guidelines on acute calcolous cholecystitis in elderly population. World J Emerg Surg..

[41] Loozen CS, Van Santvoort HC, Van Duijvendijk P (2018). Laparoscopic cholecystectomy versus percutaneous catheter drainage for acute cholecystitis in high risk patients (CHOCOLATE): Multicentre randomised clinical trial. BMJ..

[42] Castillo-Angeles M, Cooper Z, Jarman MP, Sturgeon D, Salim A, Havens JM (2021). Association of frailty with morbidity and mortality in emergency general surgery by procedural risk level. JAMA Surg..

[43] Takemoto Y, Abe T, Amano H, Hanada K, Fujikuni N, Yoshida M, Kobayashi T, Ohdan H, Noriyuki T, Nakahara M (2017). Propensity score-matching analysis of the efficacy of late cholecystectomy for acute cholecystitis. Am J Surg..

[44] Asai K, Watanabe M, Kusachi S, Matsukiyo H, Saito T, Ishii T, Kujiraoka M, Katagiri M, Katada N, Saida Y (2017). Evaluating the timing of laparoscopic cholecystectomy for acute cholecystitis in an experienced center based on propensity score matching. Asian J Endosc Surg..

